# A Case of Wellens Syndrome in a Young Adult With Intermittent Chest Pain: Understanding the Unique Pattern and Clinical Correlation

**DOI:** 10.7759/cureus.36820

**Published:** 2023-03-28

**Authors:** Oluwaremilekun Tolu-Akinnawo, Rabira R Dufera, Joseph Akamah

**Affiliations:** 1 Internal Medicine, Meharry Medical College, Nashville, USA; 2 Cardiology, Nashville General Hospital, Nashville, USA

**Keywords:** wellens syndrome, young adult, unique ecg pattern, diagnostic awareness, role of multidisciplinary approach

## Abstract

Wellens syndrome is a unique electrocardiographic (ECG) pattern usually suggestive of critical stenosis of the left anterior descending (LAD) coronary artery. Providers must recognize this pattern as it frequently occurs in symptom-free periods and represents a pre-infarction stage requiring early intervention. We present the case of a 39-year-old male with a past medical history of hypertension who was brought to the emergency department due to complaints of worsening recurrent intermittent squeezing left-sided chest pain of two months duration. Cardiac enzymes were within limits. ECG done at triage was significant for biphasic T waves in leads V2-V5 consistent with type A Wellens syndrome. The cardiology team consulted, with the patient subsequently having a percutaneous coronary intervention to the mid and proximal portion of the LAD. The patient was later discharged on the third day of admission on guideline-directed medical therapy, with plans to follow up closely with the cardiology clinic.

This case highlights the significance of using the characteristics pattern of Wellens syndrome in providing critical diagnostic and prognostic value. This article aimed to promote awareness of Wellens syndrome, the clinical correlation, and the role of timely acute management.

## Introduction

Wellens syndrome also called left anterior descending artery (LAD) T wave inversion pattern is a characteristic electrocardiographic pattern that suggests critical occlusion/thrombosis of the proximal part of the LAD coronary artery. It is a pattern that needs to be identified by providers as this determines the critical diagnostic and prognostic value [1]. Wellens syndrome was first described in 1982 after a group of patients presenting with atypical angina and subsequently having proximal LAD occlusion were found to have similar electrocardiographic patterns [2]. Although relatively common among patients with underlying cardiovascular risk factors and older age groups, Wellens syndrome remains underrecognized and undiagnosed worldwide [3-5]. Wellens syndrome is seen in about 10-15% of patients with unstable angina [5,6], with nearly three-quarters developing extensive anterior wall myocardial infarction if not recognized and managed early [7,8]. This highlights the significance of early identification of this pattern, as this may guide the prognostic value of the patients, as seen in our case.

## Case presentation

The patient is a 39-year-old male with a past medical history of hypertension, well controlled on medication, who presented to the emergency department on account of recurrent left-sided chest pain of two months duration. Left-sided chest pain was reported as squeezing in nature, intermittent with each episode lasting five minutes, occasionally mid-sternal, present both on exertion and at rest. He reported associated difficulty in breathing with each episode and an episode of diaphoresis. He, however, denied orthopnea, paroxysmal nocturnal dyspnea, palpitation, leg swelling, nausea, or vomiting. The patient’s family history is significant for myocardial infarction in the father at age 62 years. No previous/present history of tobacco/illicit drug use was reported.

**Figure 1:**
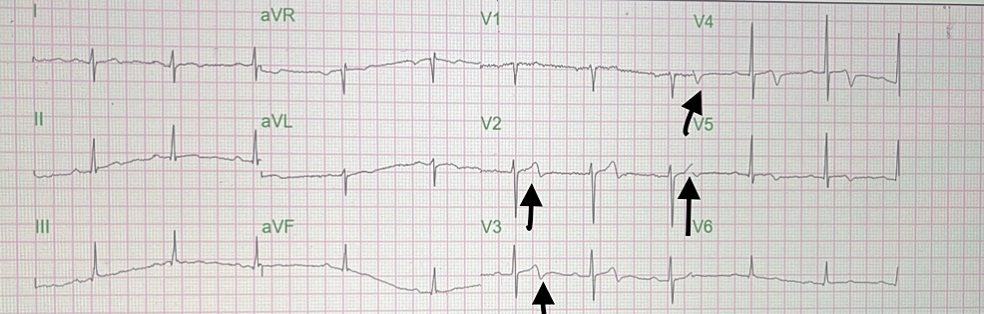
Black arrows indicate biphasic T waves in leads V2-V5.

Vital signs at presentation were unremarkable. Labs were significant for essentially normal complete blood count, comprehensive metabolic panel, and troponin level. Low-density lipoprotein (LDL) was 120 mg/dL. Urine drug tests, urinalysis, coronavirus disease (COVID), and flu testing were unremarkable. ECG was significant for biphasic T waves in leads V2-V5, consistent with type A Wellens syndrome (Figure 1).

**Figure 2:**
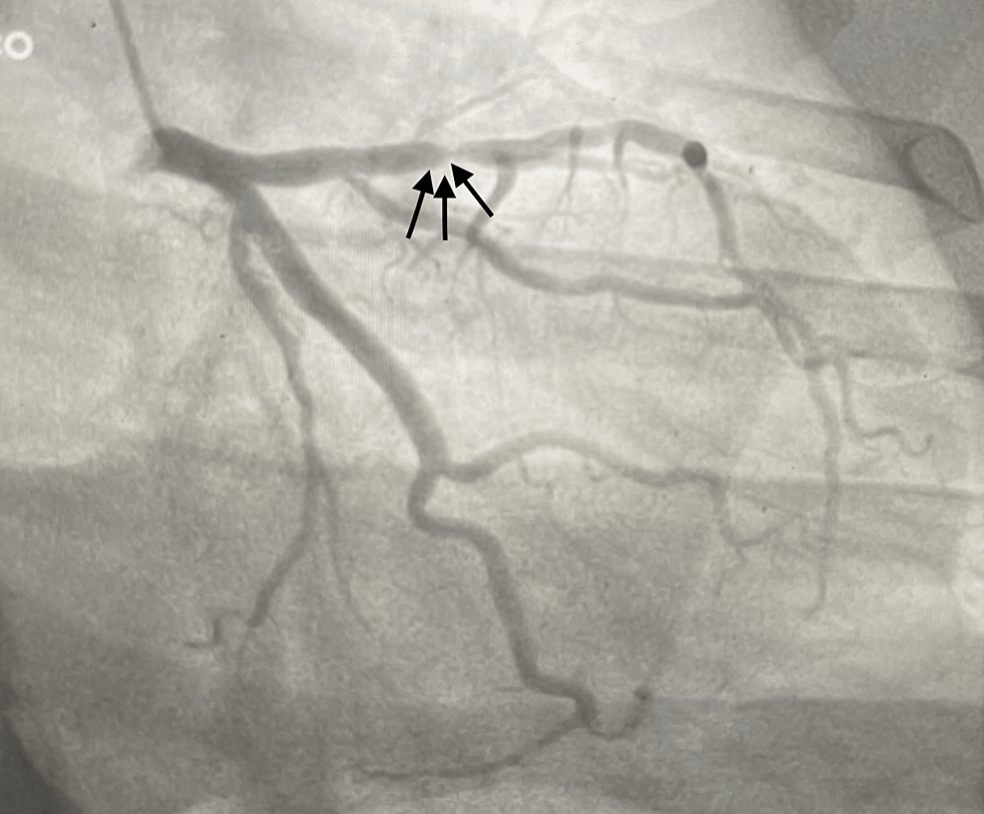
Cardiac catheterization with black arrows indicating mid-LAD stenosis. LAD: left anterior descending

**Figure 3:**
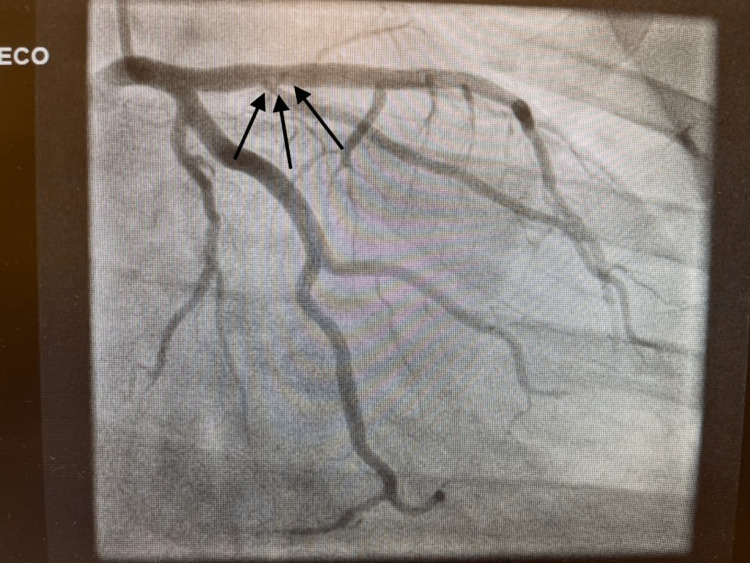
Black arrows indicating post-intervention with reversal of stenosis.

The patient was started on acute coronary syndrome protocol with a loading dose of dual antiplatelet therapy, heparin, and high-intensity statin. The cardiology team consulted with the patient, who subsequently had an urgent left heart catheterization, which was significant for a 99% mid-LAD occlusion (Figure 2). The left circumflex and right coronary artery (RCA) had minor luminal irregularities. The patient subsequently had a percutaneous coronary intervention to the mid and proximal portion of the LAD (Figure 3).

A transthoracic echocardiograph was also significant for normal left ventricular (LV) cavity size, wall motion, and global systolic function, with a visually estimated LV ejection fraction of 60-65%. The patient was continued on dual antiplatelet therapy, beta blocker, angiotensin-converting enzyme inhibitor (ACEI), and high-intensity statins without post-procedural complications. After three days of admission, the patient was discharged to follow up closely with his primary care provider and cardiology clinic.

## Discussion

When evaluating a patient with suspected myocardial infarction, understanding electrocardiographic (ECG) patterns is critical to detect active or impending myocardial ischemia. The ability to correctly detect abnormal ECG patterns is vital to early intervention and survival, and that is why every provider should be able to identify the Wellens syndrome. Wellens syndrome is a pre-infarction myocardial ischemic state of coronary artery disease (CAD), often associated with critical LAD stenosis [4]. It was first described by de Zwaan et al. in two of their studies published in 1982 and 1989 [2,7]. In these studies, characteristics ECG pattern of Wellens syndrome was present in 18% of patients, and 14% of patients admitted for unstable angina [2,7]. According to these studies, 100% of patients with this pattern had LAD stenosis, with 75% of untreated cases developing extensive anterior wall myocardial infarction [2].

With Wellens syndrome slowly gaining more attention in recent years, with more cases reported in the scientific journal, it is likely still underreported and underdiagnosed due to the lack of awareness [3-5]. This article aimed to explain the ECG criteria for Wellens syndrome, the types of patterns, and their clinical correlation, as seen in our case.

As with acute coronary syndromes, Wellens syndrome requires some suspicion, with patients sometimes presenting with atypical symptoms. However, a history of intermittent chest pain, with absent or minimally elevated cardiac enzymes, and the unique ECG pattern should raise suspicion. The ECG findings are typically notable for isoelectric or minimally elevated (<1 mm) ST segment, absent pathological Q waves, and biphasic or symmetrically deep T waves inversion in leads V2 and V3 as seen in the current case [9,10]. Several case reports have also subdivided the patterns into two types as follows: type A or B or, in other case reports, type 1 and 2 [11,12]. These have, however, been used interchangeably, which reflects some inconsistency. However, using the nomenclature initially described in the original study by de Zwaan et al., type A is the less common pattern with biphasic T waves, as seen in this case. In contrast, the type B pattern is the symmetrical deep T wave inversion in leads V2 and V3 [2,7]. It is also worth knowing that although type A is less common (approximately 24%), it is associated with higher fatality [13].

It is well established that Wellens syndrome indicates a pre-infarction state of myocardial infarction, early recognition of the pattern and involvement of the cardiology team is critical to survival. This is because an urgent coronary angiography rather than a stress test is needed to identify the lesions and correct them as needed [14]. A cardiac stress test may provoke ischemia by increasing myocardial demand, inducing ST-elevation myocardial infarction, left ventricular failure, life-threatening arrhythmias, such as ventricular tachycardia, and even death [9]. The early recognition of the pattern and involvement of the cardiology team was crucial to our patient’s survival, as the urgent cardiac catheterization revealed a critical LAD stenosis which was repaired. Late recognition/miss could have been potentially fatal in the case of our patient.

While Wellens syndrome is often linked to LAD stenosis, it is also worth knowing that these patterns can be seen in a few other clinical scenarios. They can be seen in Takotsubo cardiomyopathy, drug-induced cardiac injury from cocaine/phencyclidine/cannabis/morphine overdose, congenital myocardial bridge, and acute cholecystitis [15]. These scenarios are called pseudo-Wellens syndrome. This highlights the significance of involving the cardiology team in any case of suspected Wellens pattern, as their recommendations can be valuable in avoiding potentially invasive procedures that are not needed.

This case also highlights another case of acute coronary syndrome - Wellens syndrome in a young adult, with an estimated 6% of young adults (<45 years) contributing to mortality from CAD globally [16-18]. Therefore, suspected Wellens syndrome should follow the same management algorithm regardless of age.

## Conclusions

Although recently gaining some attention, Wellens syndrome remains underrecognized and missed. This is partly due to computerized misinterpretation as non-specific T wave abnormalities on ECG and sometimes from lack of awareness on the part of the providers. Wellens syndrome often represents a pre-infarction state of myocardial infarction and, if missed, could lead to potentially fatal outcomes. Every provider needs to be familiar with the ECG pattern and clinical presentation, as early recognition and management are critical to the patient's survival. With this case highlighting another case of acute coronary syndrome in a young adult, risk stratification needs to be done early, with appropriate preventive strategies introduced as soon as possible.
